# The synthesis of functionalized bridged polycycles via C–H bond insertion

**DOI:** 10.3762/bjoc.12.97

**Published:** 2016-05-17

**Authors:** Jiun-Le Shih, Po-An Chen, Jeremy A May

**Affiliations:** 1 Department of Chemistry, University of Houston, 3585 Cullen Blvd., Fleming Bldg. Room 112, Houston, TX 77204-5003, United States

**Keywords:** bridged rings, carbene, cascade reaction, C–H bond insertion, nitrene

## Abstract

This review presents examples from the chemical literature of syntheses of bridged-polycyclic products via C–H bond insertion by carbenes and nitrenes. Applications to natural product synthesis, a description of the essential elements in substrate-controlled reactions, and mechanistic details of transformations are presented. Overall, these transformations allow the construction of important ring systems rapidly and efficiently, though additional catalyst development is needed.

## Introduction

**Figure 1:**
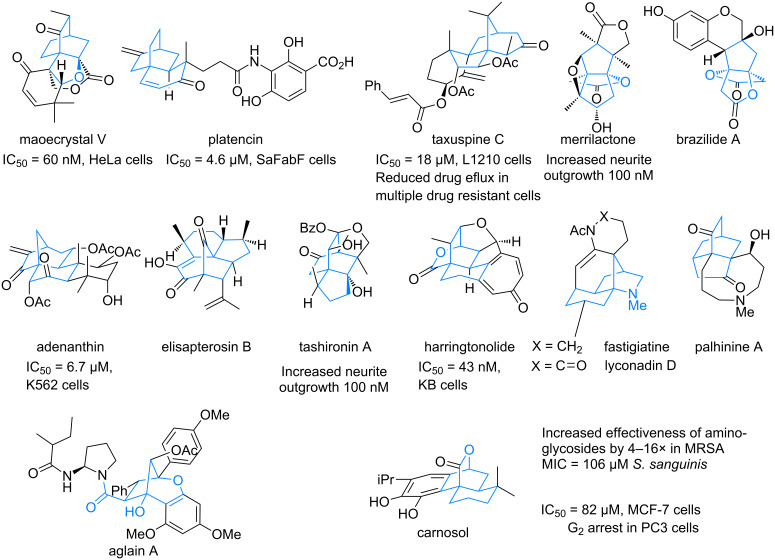
Bridged polycyclic natural products.

Bridged polycyclic natural products are an inviting challenge to the synthetic chemist for their rich display of functional groups, densely packed structures, and inherent architectural three-dimensionality. Many of these compounds exhibit biological activity that is potentially useful (Figure 1) [1–12]. Of these, some exhibit exquisite selectivity for a single target, presumably because their rigid structures restrict molecular conformations for binding to multiple biological entities. Given the natural scarcity of most of these compounds, few have been extensively screened for biological activity, and some have not been tested at all.

**Figure 2:**
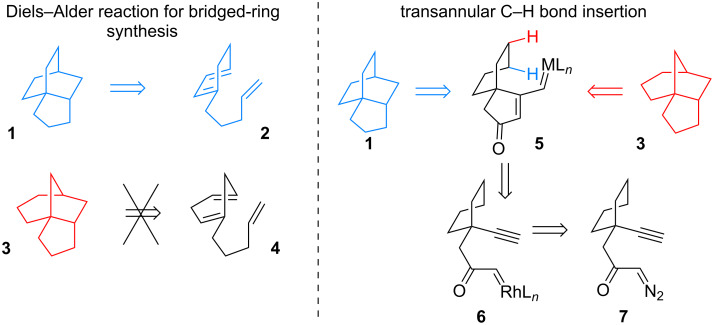
Strategic limitations.

Unsurprisingly, many synthetic strategies for the construction of bridged rings have been reported. These methods usually rely on existing functional groups to build the rings, consequently leading to the synthesis of a single isomer. To access a different isomer, either the synthetic sequence must be revised to install the key functional groups at alternative positions, or the synthetic strategy must be completely altered as the original method is not capable of synthesizing other isomers. For example, multiple research groups have shown that the bridged bicyclo[2.2.2]octane core **1** of maoecrystal V may be constructed via an intramolecular Diels–Alder reaction from a functionalized 1,3-diene like **2** (Figure 2) [13–16]. However, such a reaction would not be applicable to synthesize the bicyclo[3.2.1]octane core **3** of taxuspine C, as the mechanistic requirements in a Diels–Alder cycloaddition are not met with a 1,4-diene.

Strategies that may access multiple structural isomers of bridged polycycles from a common intermediate offer multiple advantages: saved time and effort by avoiding the validation of new strategies for each target, accumulated understanding of the key reactions in the strategy for higher yields and improved efficiency, and rapid access to multiple targets from a single intermediate produced on large scale that may be stored until needed [17]. The C–H bond insertion has great potential as a method to access different polycyclic isomers (e.g., **1** or **3**) through C–C or C–N bond formation from a carbene or nitrene, respectively, without having to preinstall functional groups at those positions (Figure 2). Ideally, the choice of catalyst or reagent would control the product formed. The transformation of **5** to **1** or **3** could occur in a single-bond-forming event, or multiple rings could be synthesized in a cascade reaction (i.e., **6** to **1** or **3**).

**Scheme 1:**
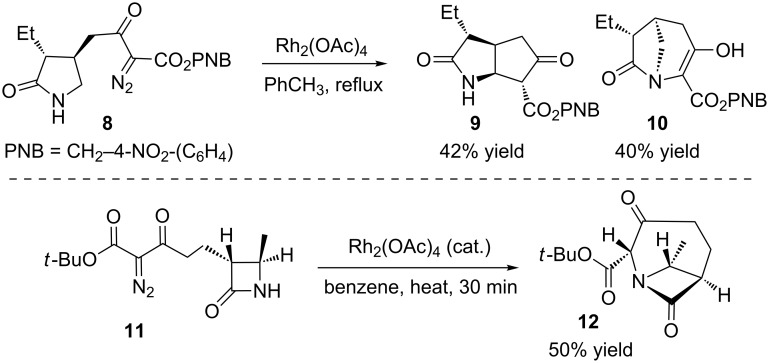
Bridged rings from N–H bond insertions.

This review highlights strategies that leverage C–H bond insertion by carbenes and nitrenes to construct bridged polycycles. This key bond-forming event may construct the bridged ring, or it may construct an additional ring fused to an existing bridged bicycle to synthesize the bridged-polycyclic product. While some examples of bridged-azacycle formation via N–H bond insertion are known (Scheme 1) [18–19], they are less common. We note that the development of synthetic methods to access the goal portrayed in Figure 2 is still in the early stages, with most of the work discussed having been reported within the last decade. Taken together, this work has elucidated many of the substrate factors that control the reaction. The development of catalysts to control isomer formation has yet to be achieved, though it is our hope that this review will inspire efforts for their development. We have organized this review by the catalyst used, with free carbenes first, followed by Cu, Rh, Au, Pt, and then W-catalyzed reactions.

## Review

### Metal-free reactions

**Scheme 2:**
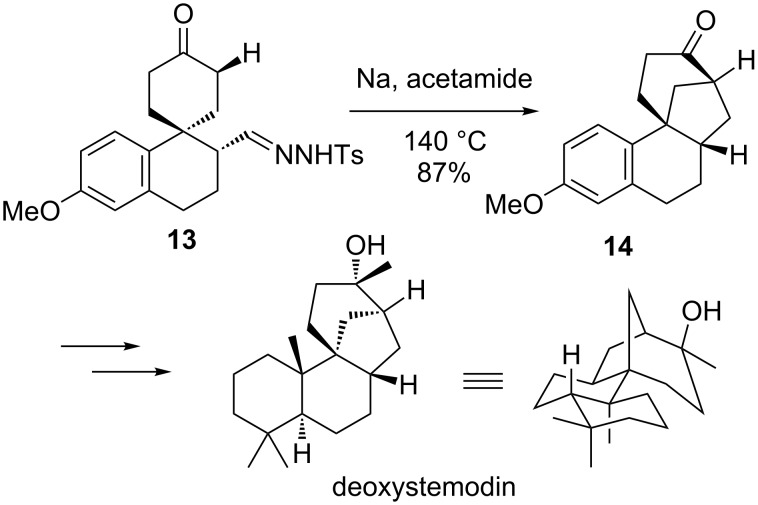
The synthesis of deoxystemodin.

While transition metal catalysis has seen widespread adoption for carbene and nitrene reactions, it is not necessary for a controlled reaction in all cases. Chatterjee reported an early example of building bridged-polycyclic intermediates using metal-free carbenes in 1979 (Scheme 2) [20]. Exposing the tosylhydrazone **13** to base and heat generated an alkyl carbene that inserted into the C–H bond of an adjacent ring. Surprisingly, the insertion reaction was more rapid than a 1,2-hydride shift or rearrangement, and the bridged product **14** was obtained in 87% yield. This compound was advanced to synthesize the natural product deoxystemodin [21–25].

**Scheme 3:**
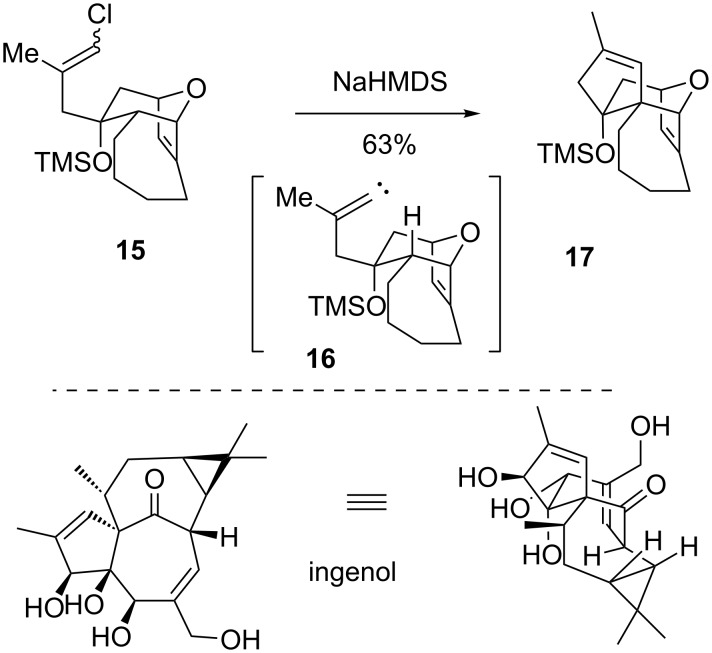
A model system for ingenol.

Grainger reported an approach to synthesize the bridged core of ingenol via C–H insertion (Scheme 3) [26]. Here, an existing bridged ring with a pendant vinyl chloride was synthesized (see **15**). The addition of base promoted the formation of a vinylidene carbene **16**, which then inserted into the more electron-rich methine C–H bond to generate the fused cyclopentenyl ring in **17**. While this intermediate was not advanced in a total synthesis of the natural product, it demonstrates the potential of the strategy. Additionally, the reaction was described to proceed through the lowest energy conformation of the carbene intermediate **16**, but an extensive study of conformational effects was not reported.

**Scheme 4:**
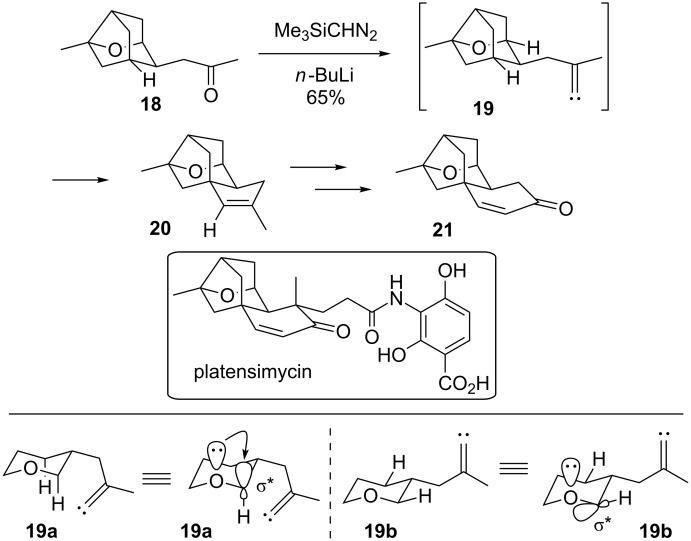
Formal synthesis of platensimycin.

More recently, Lee showed that vinylidene carbenes could be used to build the ring-fused bridged system **21** found in platensimycin (Scheme 4) [27]. The vinylidene carbene **19** preferentially inserted into the C–H bond further from the oxygen, which is contrary to many intramolecular studies that have found that heteroatoms accelerate C–H bond insertion at adjacent carbons. The Lee group performed further studies on the electronic effects of the oxygen on the rate of bond insertion, and concluded that the ability of the oxygen lone pair to align with the σ* orbital of the C–H bond targeted for insertion (see illustration **19a**) is required for rate acceleration. Equatorial C–H bonds adjacent to endocyclic oxygens cannot achieve the correct orbital alignment (see illustration **19b**), and so the electronegativity of the oxygen actually deactivates insertion into the equatorial C–H bond. This is the case for carbene **19**, since the alkyl bridge locks its conformation in such a way that prevents an activating alignment, and the oxygen acts as an inductive electron-withdrawing group. The alkene in the cyclopentene of **20** was oxidatively cleaved, and then an aldol condensation gave the cyclohexenone **21**, which was an intermediate in Nicolau’s platensimycin synthesis [28].

**Scheme 5:**
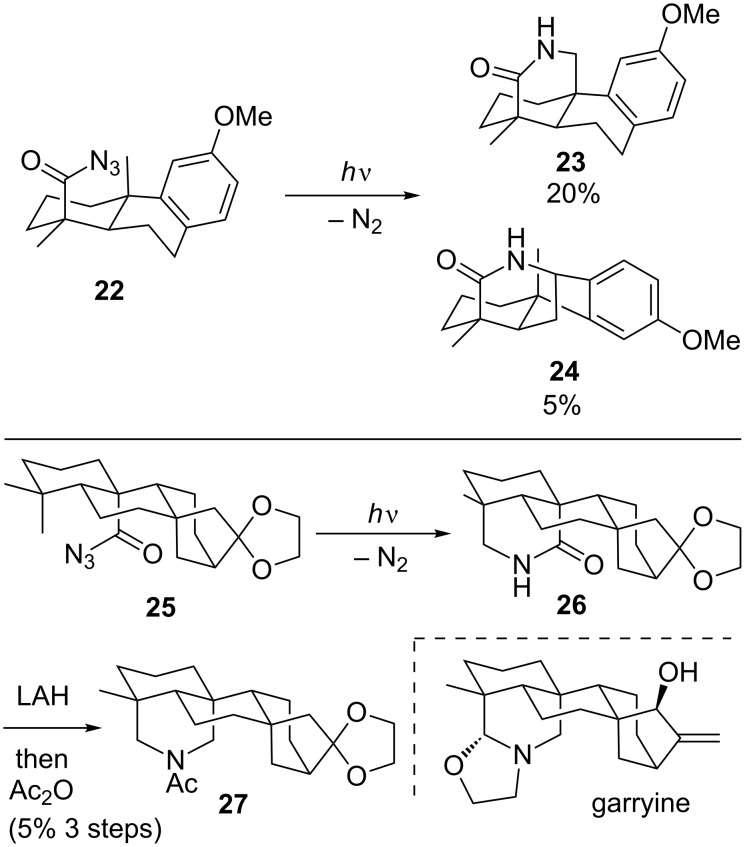
The formal synthesis of gerryine.

Carbenes are not the only species that have been used to build bridged polycycles without metal catalysts. Masamune demonstrated that the nitrene generated from the acyl azide **22** via ultraviolet irradiation inserted into a nearby methyl group to give **23** (Scheme 5) [29–30]. Competition with a transannular benzylic C–H insertion to give **24** was a minor outcome. This initial study provided a model system to confirm the proposed stereochemistry of atisine. A later study using the substrate **25** that lacked the benzylic C–H bond led to a formal total synthesis of garryine, which is closely related to atisine. The initial lactam **26** that was formed by methyl C–H insertion was quite unstable, and so it was quickly converted to the acetamide **27**. Advancing that intermediate intercepted a route to garryine completed by Pelletier [31].

### Metal-catalyzed reactions

#### Copper

**Scheme 6:**
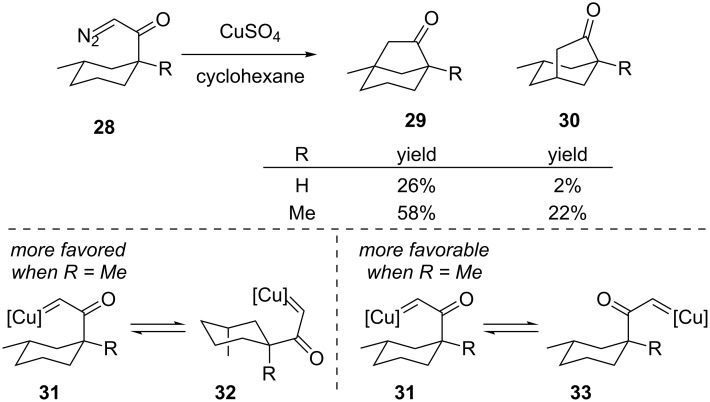
Copper-catalyzed bridged-ring synthesis.

The earliest example to our knowledge of transition metal catalysis for the formation of bridged rings via C–H bond insertion was Wolff’s use of copper (Scheme 6) [32–33]. Silver was also examined as a possible catalyst, but then a Wolff rearrangement was the primary outcome. The major product with copper was insertion into the more electron-rich methine to give bicyclo[3.2.1]octane **29**. The yields of both transannular C–H insertion products were increased relative to the Wolff rearrangement when a methyl group was present on the cyclohexyl carbon bearing the diazoketone (**28**, R = Me). There are two reasons this methyl substituent affects the reaction: firstly, it helps favor a ring conformation where the copper carbene derived from the diazoketone is axially disposed (see **32**) and thus is closer to the C–H bonds for insertion. Secondly, the methyl group helps bias the rotation about the ketone–cyclohexyl bond so that the carbene may be conformationally disposed over the ring as shown in **31** instead of the exocyclic conformation **33**.

#### Rhodium

**Scheme 7:**
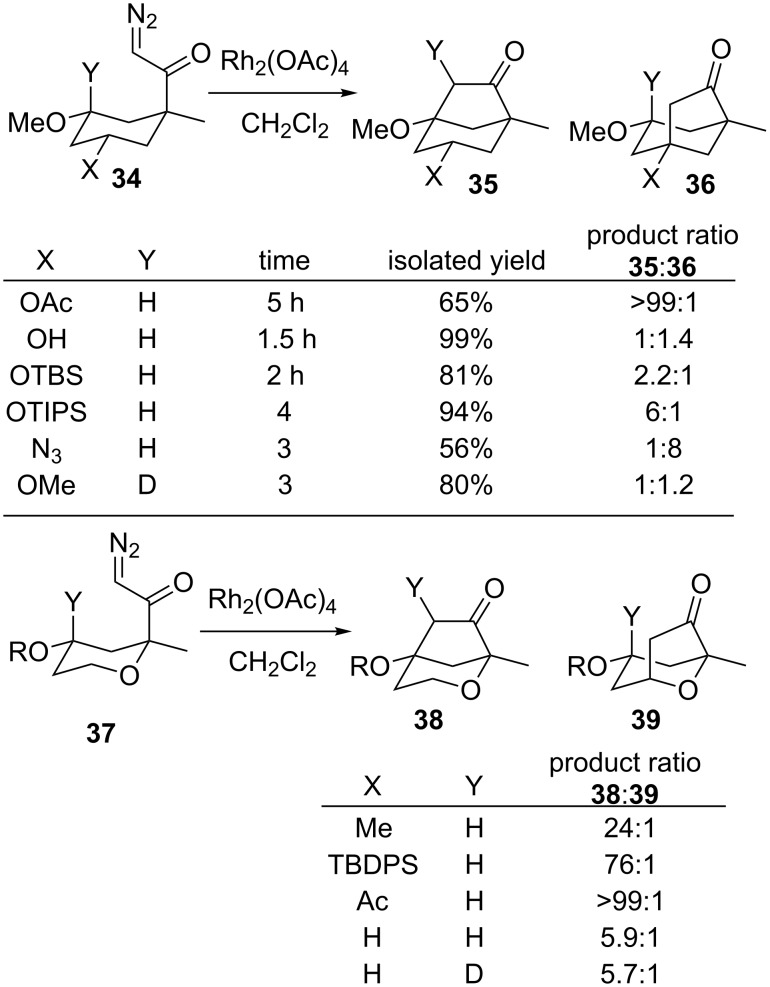
Factors influencing insertion selectivity.

The most common metal seen in C–H bond insertions for the formation of bridged rings is rhodium. Adams used Rh_2_(OAc)_4_ to further develop Wolff’s early work to determine selectivity patterns for transannular insertions (Scheme 7) [34–35]. His group examined the effects of heteroatom substituents such as ethers and an azide in the 3-methoxycyclohexyl substrate **34**. The observed patterns largely mirrored those found in intramolecular C–H bond insertions to form monocyclic and fused bicyclic rings [36–59]. The stronger the electron donor, the greater the observed insertion at the C–H bond on the carbon with that donor. For example, insertion at the methoxy-substituted carbon to give **35** (X = OAc) occurred more rapidly than at an acetoxy-substituted carbon to form **36**. Sterics also seemed to play a role, as insertion near a TIPS ether in **36** (X = OTIPS) was less than that for a TBS ether (**36**, X = OTBS) [60]. A hydroxy group had a similar effect to the methoxy substituent, and the azide was the only group to activate the C–H bond more than a methoxy group. Interestingly, the endocyclic oxygen in substrate **37** was not nearly as effective at activating the adjacent C–H bond for insertion to give **39** as the exocyclic oxygen was to provide **38**, even when the exocyclic oxygen was acetylated. This is likely related to the effects observed by Lee (Scheme 4). The Adams group also looked for a deuterium kinetic isotope effect for insertion in both **34** (Y = D, X = OMe) and **37** (Y = D, R = H). Only a small difference in relative rates between C–H and C–D bond insertion was seen for either substrate (e.g., compare **35** to **36**, Y = D, X = OMe). Adams proposed that a late transition state must be operative for insertion, and so the isotope effect was not pronounced.

**Scheme 8:**
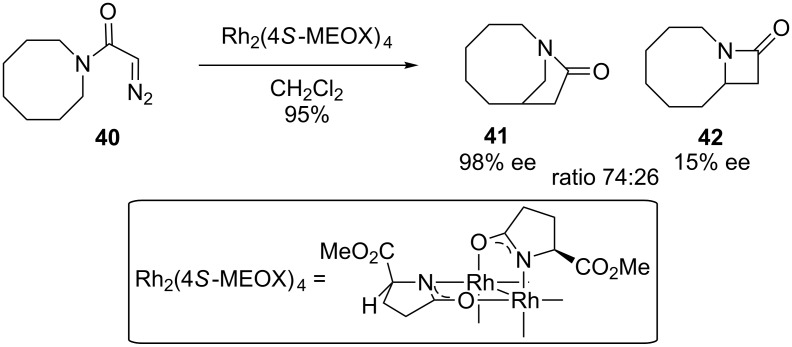
Bridged-lactam formation.

Doyle expanded the scope of the potential bridged products through the use of diazoacetamides (Scheme 8) [61]. He found an important example of a transformation where the ratio of gamma vs beta-lactam formation could be controlled to a significant extent by catalyst choice. In particular, Rh_2_(4*S*-MEOX)_4_ favored the bridged γ-lactam **41** over the fused β-lactam **42** in about a 3:1 ratio. The conformation of the cyclooctyl ring may also play a role in the selectivity as discussed below.

**Scheme 9:**
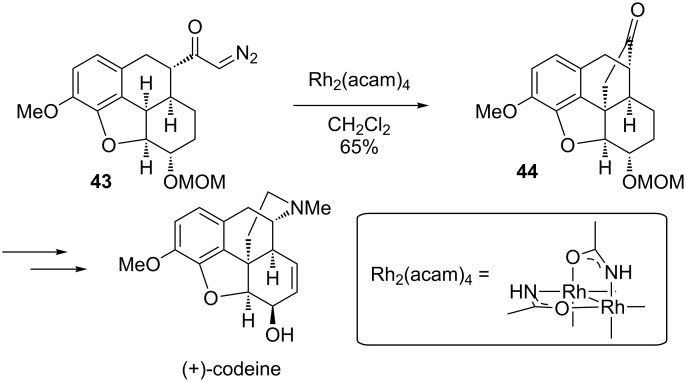
The total synthesis of (+)-codeine.

The White group used rhodium dimers as catalysts to form the central quaternary carbon of (+)-codeine (Scheme 9) [62]. This insertion into the benzylic methine of **43** was quite selective, with only a single reported product. The formation of this hindered carbon stereocenter could be potentially quite difficult otherwise. While a slightly better yield for the insertion was obtained if the OMOM ether was replaced with a ketone functional group, that product was problematic in attempts to advance it to codeine. It is notable that the intramolecular C–H bond insertion to form the bridged polycycle was significantly faster than an intermolecular insertion into the MOM acetal methylene, which would be electronically activated by the two flanking oxygen substituents.

**Scheme 10:**
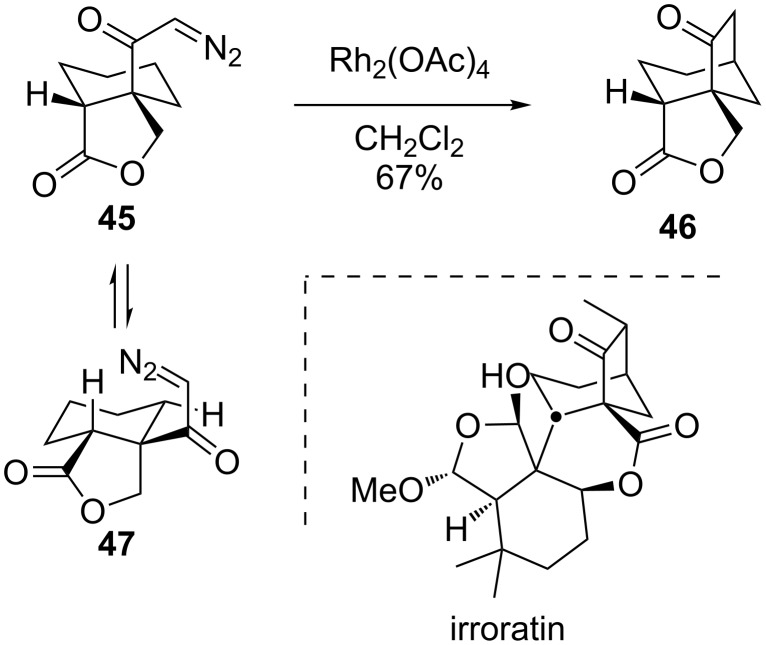
A model system for irroratin.

Later, Magnus showed that a conformationally flexible fused bicycle like **45** was also viable as a substrate for bridged-ring synthesis (Scheme 10) [63]. While a conformational flip to the other chair form **47** is perhaps more favored energetically, the insertion into the proximal C–H bonds in **47** would lead to a cyclobutanone, which is apparently slower than the ring flip and transannular insertion by an axially disposed ketocarbene into an axial C–H bond. Magnus noted that **46** bears a striking resemblance to the bridged polycyclic lactone core of irroratin and proposed that this method could be used for its synthesis.

**Scheme 11:**
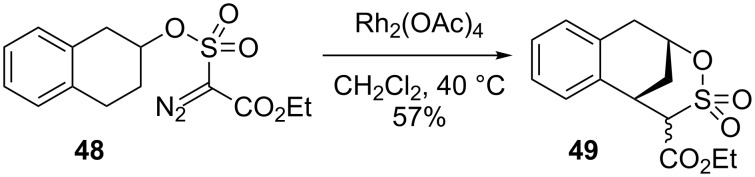
The utility of 1,6-insertion.

The discovery by Du Bois that carbenes and nitrenes generated from sulfonate esters prefer 6-membered ring formation (i.e., 1,6-insertion) opened the door for easy access to 1,3-functionalized products via C–H insertion [64–65]. The sulfonate can be a useful functional group for subsequent transformations, also. If used in the presence of an attached ring as in **48**, the 1,6-insertion produced the bridged-bicyclic product **49** (Scheme 11) [66].

**Scheme 12:**
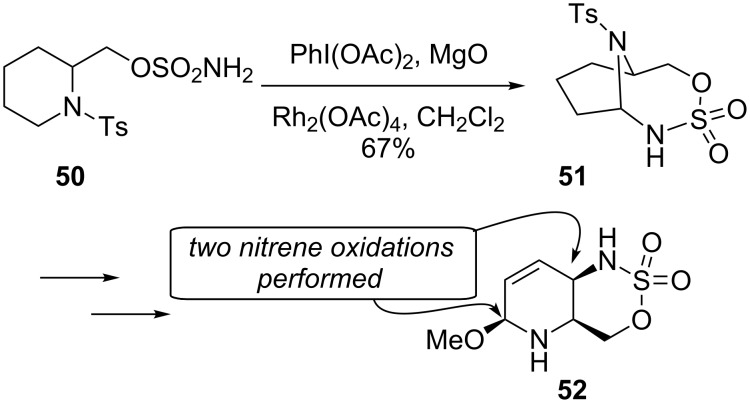
Piperidine functionalization.

The Compain group leveraged this innovation in the synthesis of functionalized piperidines (Scheme 12) [67]. By using the piperidine **50**, an initial insertion into the amine-activated C–H bond generated the tosylamine-bridged bicycle **51**. The aminal in **51** could be transformed to a methyl hemiaminal, and then later a second C–H bond insertion by another nitrene targeted the less activated C–H bond to form the ring-fused piperidine **52**. Thus, the pendant sulfonamide acted as a tool for multiple remote functionalizations on the piperidine ring. Many examples of the sequence with different piperidines were also reported. The resulting 3-amino-2,6-disubstituted piperidines like **52** are known to display anti-allergic and anti-inflammatory activities.

**Scheme 13:**
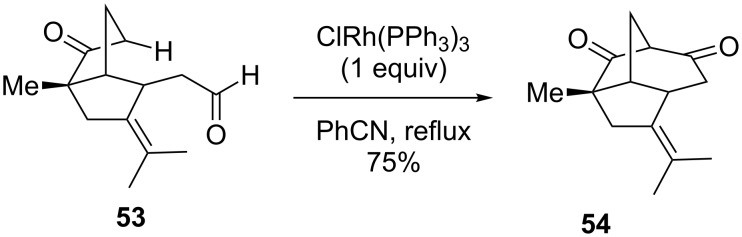
Wilkinson’s catalyst for C–H bond insertion.

Rao generated a bridged polycycle via a formal C–H bond insertion using Wilkinson’s catalyst (RhCl(PPh_3_)_3_) instead of the usual rhodium(II) dimer (Scheme 13) [68]. Rather than starting with a diazoketone, ester, or amide, Wilkinson’s catalyst may generate an active organorhodium intermediate through insertion into the acyl C–H bond of the aldehyde **53**. A subsequent transannular C–H functionalization at the site of a weakly acidic proton then produces the bridged product **54**. While an innovative transformation, it should be noted that a full equivalent of Wilkinson’s catalyst is needed as there is no catalytic turnover observed.

**Scheme 14:**
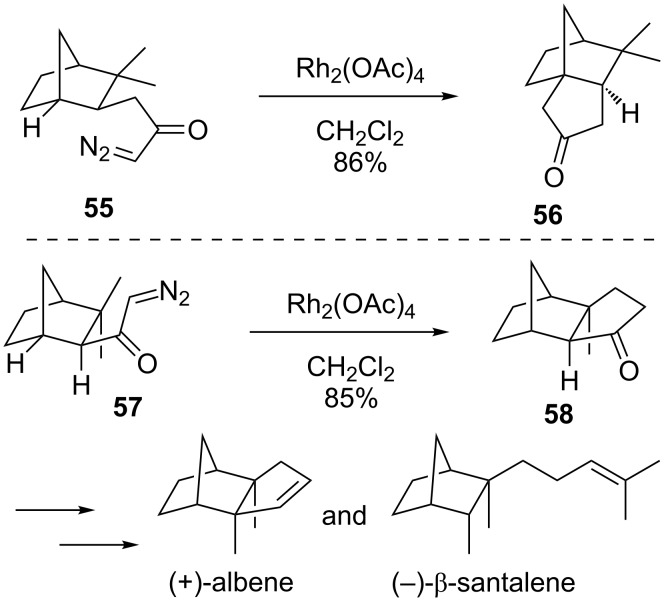
Bridgehead insertion and the total synthesis of albene and santalene.

Bridged polycycles may also be accessed synthetically by the formation of a new ring on an existing bridged bicycle via C–H bond insertion. Early results for this approach using rhodium(II) dimers were disclosed by Sonowane [69–70]. He found that the tether length of the diazoketone to the bridged ring had a profound effect on the preferred C–H insertion location (Scheme 14). As has been noted for other intramolecular insertions [42–65], five-membered ring formation is generally preferred over other ring sizes. Thus, the diazopropanone-substituted norbornane **55** saw insertion into the bridgehead C–H methine to generate the cyclopentanone-fused norbornane **56**. However, the shorter tether in diazoethanone-substituted **57** would form a cyclobutanone-fused product if bridgehead insertion occurred, and so insertion into a methyl C–H bond was preferred to give a different cyclopentanone-fused norbornane, **58**. This latter intermediate was used in the total syntheses of (+)-albene and (−)-β-santalene.

**Scheme 15:**
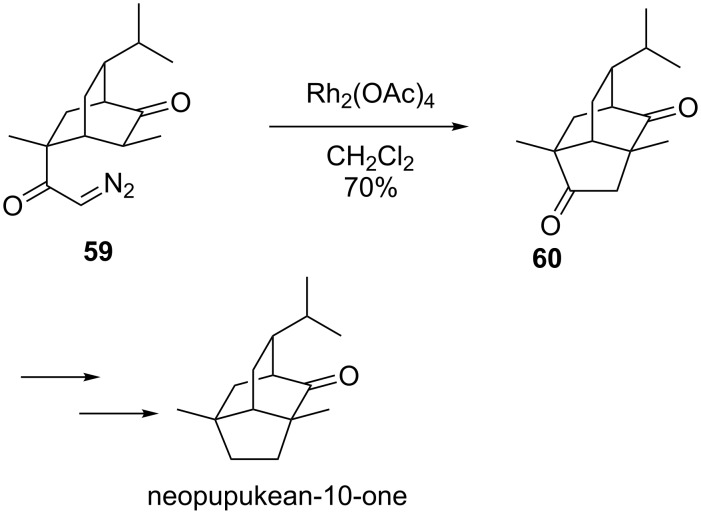
The total synthesis of neopupukean-10-one.

Srikrishna found that caged polycycles could be similarly formed via a transannular insertion [71–75]. His group synthesized isotwistanes (2)-neopupukean-4,10-dione, (2)-neopupukean-10-one (shown), 2-thiocyanatoneopupukeanane, (−)-2-pupukeanone, (−)-4-thiocyanatoneopupukeanane, and (±)-9-isocyanoneopupukeanane from carvone all with a nearly identical strategy to that in Scheme 15.

**Scheme 16:**
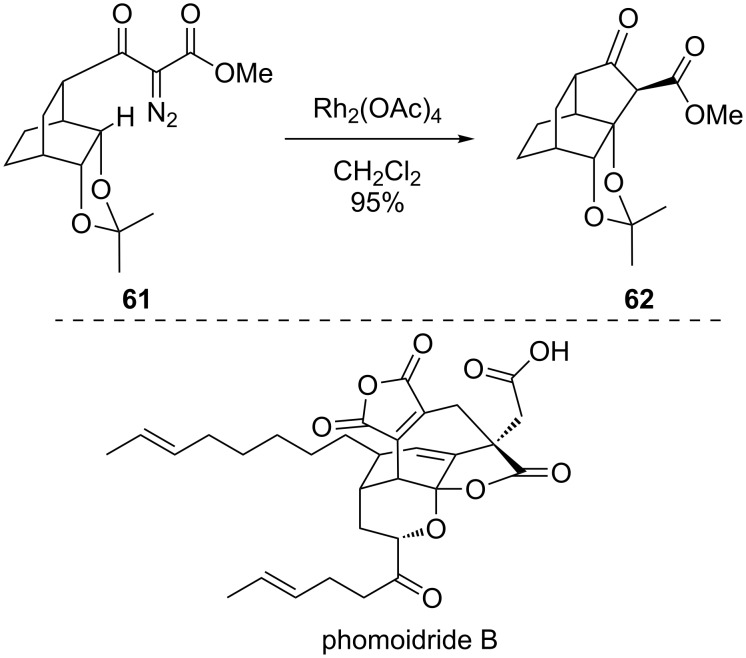
An approach to phomoidride B.

The Wood group applied a similar strategy toward the synthesis of phomoidride B (Scheme 16) [76]. While this strategy was ultimately not productive for the total synthesis of the natural product, it did synthesize the key synthetic intermediate **62** very rapidly by taking advantage of insertion into the ether-activated C–H bond of **61**.

**Scheme 17:**
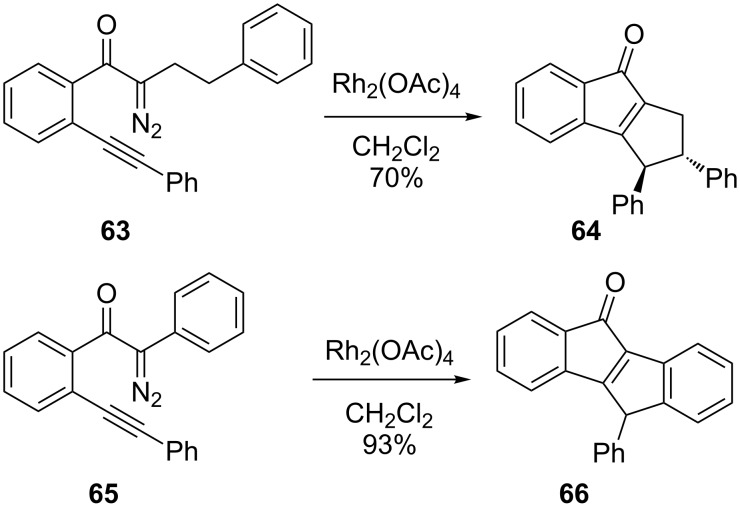
Carbene cascade for fused bicycles.

A carbene cascade reaction coupled with C–H bond insertion offers the potential to build both the bridged bicycle and additional fused rings in a single reaction (see Figure 2). The foundation of this strategy was laid by Hoye and Padwa [77–85]. Some substrates with a well-defined C–H bond insertion as the final step were reported, though these generally produced fused polycycles (Scheme 17) [86].

**Scheme 18:**
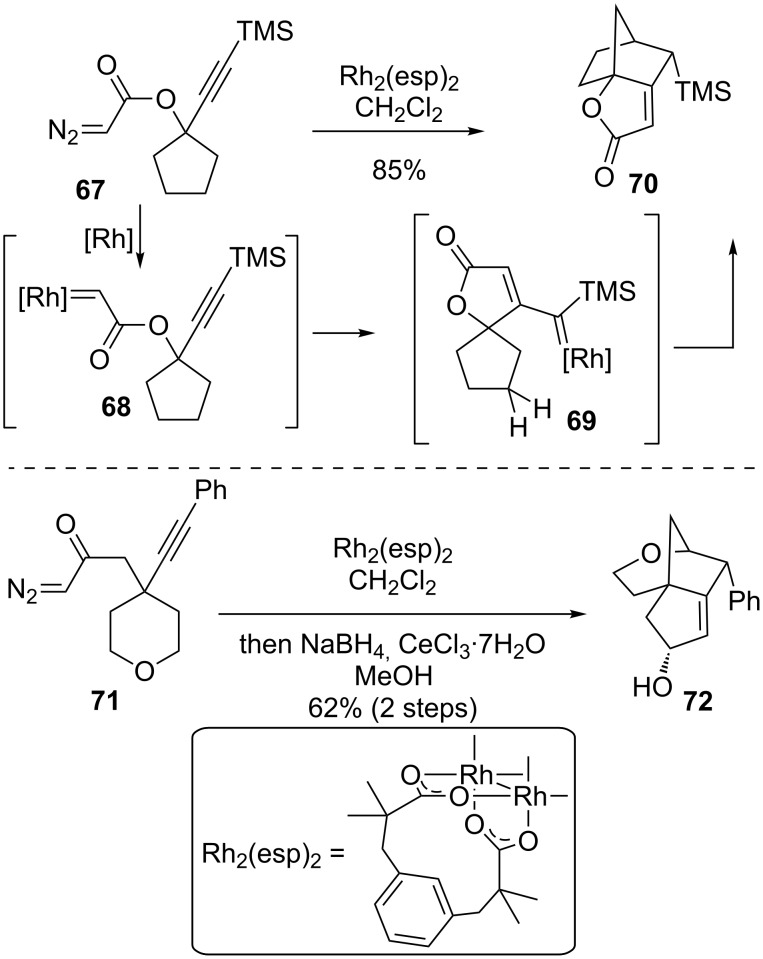
Cascade formation of bridged rings.

The May group took advantage of this cascade to synthesize bridged polycycles from monocyclic precursors [87–89]. Both diazoesters and diazoketones were examined for their potential to initiate the cascade (Scheme 18). While the exact mechanistic intermediates for this transformation have not been conclusively defined [90], the reaction may be thought of as proceeding through an initial dediazotization to form a rhodium carbene, **68**, reaction with the alkyne to generate the butenolide carbene **69** or its reactive equivalent, and then C–H insertion into a cyclopentyl methylene. Insertion into a methylene adjacent to the spirocyclic center in **69** would create a highly strained spirocyclic cyclobutane ring having an sp^2^ carbon center. Thus, the bicyclo[2.2.1]heptane **70** is the major product, but a sterically hindered catalyst such as Rh_2_(esp)_2_ or Rh_2_(OPiv)_4_ is needed to prevent dimerization of the diazoacetate. These catalysts are presumed to protect the carbene intermediates from intermolecular reactions long enough to adopt the correct conformation for bridged-ring formation.

**Scheme 19:**
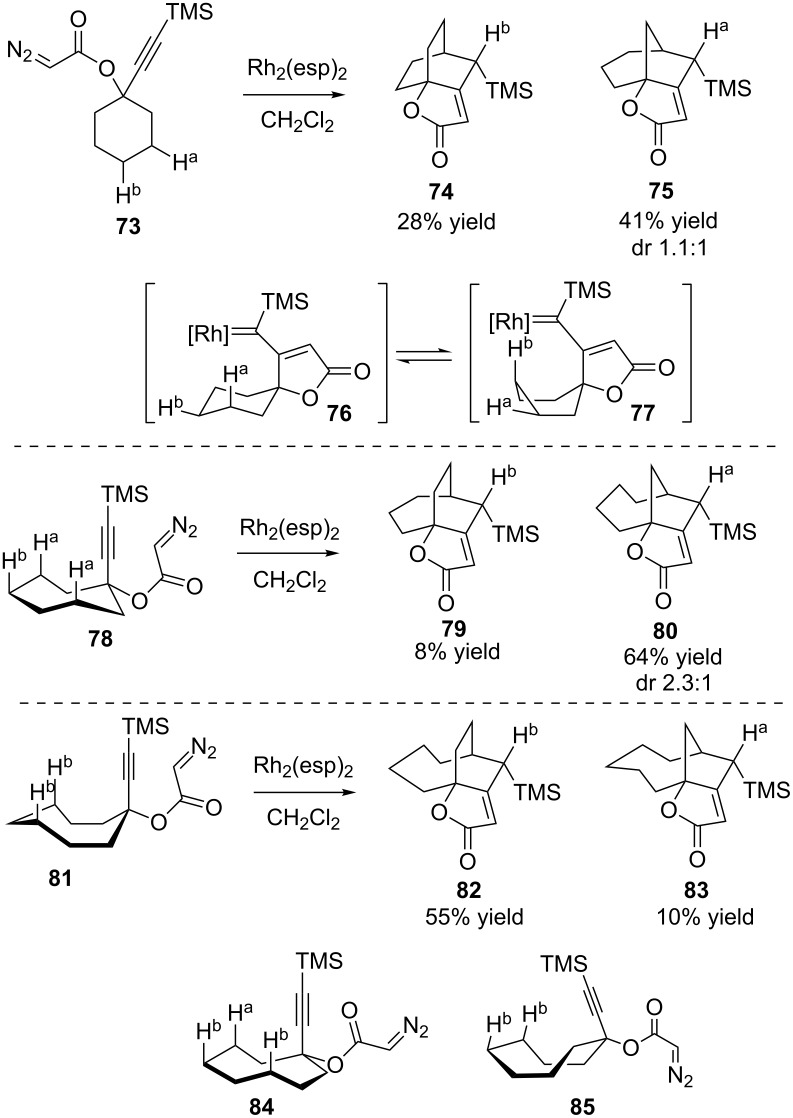
Conformational effects.

An exploration of the effect of substrate ring sizes demonstrated that the substrate conformation has as much control for insertion selectivity as electronics and size of the ring being formed. The conformational flexibility of the cyclohexyl ring in **73** led to a product ratio that roughly corresponded to the prevalence of the two non-equivalent methylenes available for insertion, suggesting that the barriers for insertion into the 3 position from conformation **76** was similar to that for the 4 position from conformation **77** (Scheme 19). This similarity in rates makes the substrate **73** suitable as a test case to find a catalyst to control the product isomer formed in the reaction as envisioned in Figure 2. The cycloheptyl and cyclooctyl rings showed a stronger intrinsic product isomer preference, however. Assuming that the carbene (or reactive equivalent) generated from the alkyne after butenolide formation (see **76**) will need to be in an axial orientation for a transannular C–H bond insertion, the lowest energy ring conformation **78** may be drawn for the cycloheptyl ring [91] with the alkyne representing the reactive carbene. The depiction of conformation **78** shows that the most accessible C–H bonds are likely to be C–H^a^ at the 3 position. The observed product distribution supports this analysis, as the bicyclo[4.2.1]nonane **80** is the major product. A similar analysis for the cyclooctyl substrate leads to **81** as the most likely conformation [91]. Here, the most available C–H bonds for insertion would give bicyclo[4.2.2]decane **82**, which would require the formation of a new 6-membered ring instead of a 5-membered ring, though the latter is typically preferred in intramolecular reactions [36–59]. Nevertheless, **82** was produced as the major product, with the minor product coming from the predicted second-most preferred conformation **84**.

**Scheme 20:**
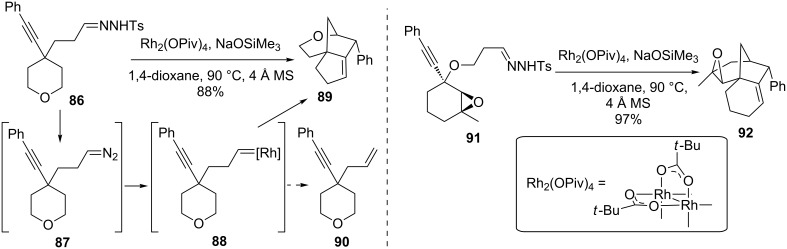
Hydrazone cascade reaction.

The May group also showed that the cascade reaction could be initiated from hydrazones. In the course of this work, it was discovered that NaOSiMe_3_ was a superior base for hydrazone to diazo conversion (i.e., **86** to **87**, Scheme 20) [89]. Surprisingly, no reaction was observed in the absence of the Rh catalyst, suggesting that it may be involved in the transformation of the hydrazone to **87**. While the intermediate alkyl carbene **88** could potentially undergo a 1,2-hydride shift to give the alkene **90** in a Bamford–Stevens-like transformation, the reaction with the adjacent alkyne proved to be much faster to provide the bridged-polycyclic product **89**. The conditions employed were sufficiently mild and chemoselective that the epoxide in cyclohexane **91** remained intact in the reaction to form **92**.

**Scheme 21:**
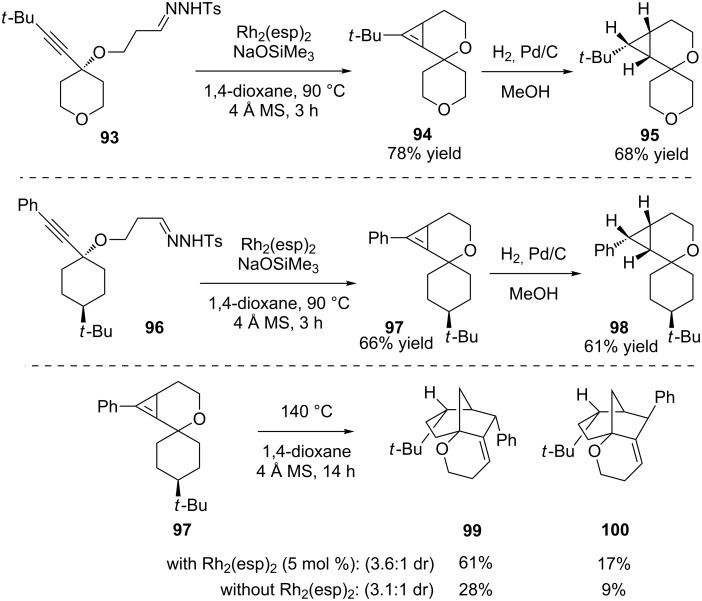
Mechanistic studies.

The use of substrates with a 3-atom tether to the hydrazone and sterically large substituents allowed the isolation and characterization of mechanistic intermediates from the cascade reaction when it was conducted at 90 °C (Scheme 21). This confirmed some prior proposals of a cyclopropene intermediate [92], as the cyclopropene **97** reacted [93–94] to form the same products as the hydrazone **96** did directly when heated to 140 °C.

#### Gold

**Scheme 22:**
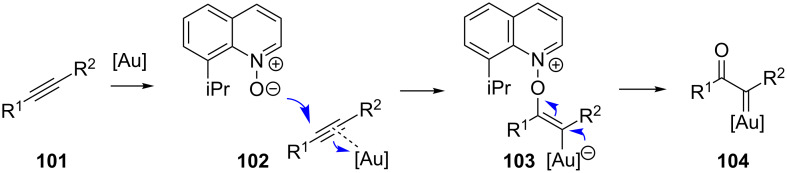
Gold carbene formation from alkynes.

The work by the May group was soon followed by gold-promoted carbene/alkyne cascades. These cascades rely on Zhang’s discovery that the use of pyridine *N*-oxides allow for the formation of gold ketocarbenes **104** from alkynes (Scheme 22) [95]. Those carbenes are then capable of further transformations, including C–H bond insertion and the reaction with other alkynes. Notably, this approach avoids the use of unstable diazo compounds.

**Scheme 23:**
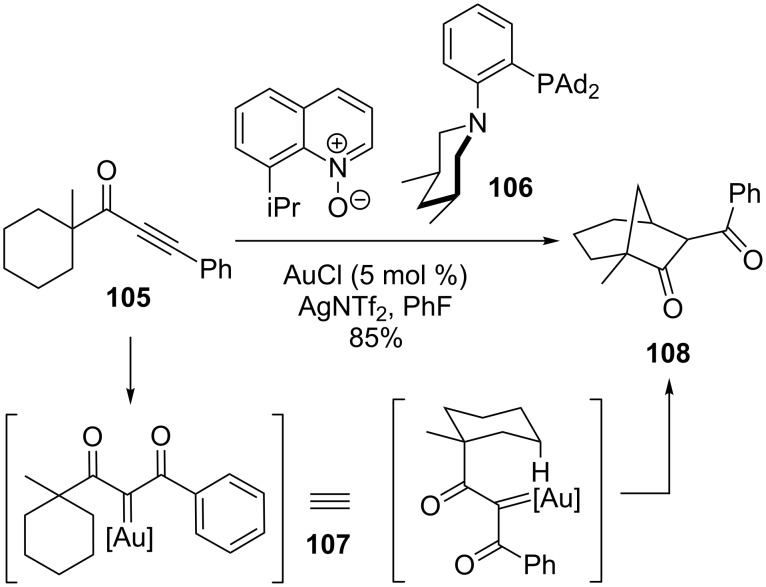
Au-catalyzed bridged-bicycle formation.

The Zhang group also demonstrated that the gold carbenes generated from alkynes can produce bridged bicycles from C–H bond insertion (Scheme 23) [96]. This was first implemented by transannular insertion via **107** to give the bicyclo[3.2.1]octane **108**. The diastereoselectivity of the process was not reported. Again, dual gold catalysts were used with a pyridinium oxide oxidant. Notably, a hindered ligand was again necessary for bridged-bicycle formation – in this case the diadamantyl phosphine **106**. The Thorpe–Ingold effect was also found to be highly beneficial for the reaction. An interesting mechanistic study using a less selective substrate showed that the same array of products could be obtained from either an alkynyl ketone or the corresponding diazoketones. The product distribution was the same for either starting material, though the latter took 4 days to go to completion instead of 2 hours. Unlike the studies by Adams (Scheme 7), a significant deuterium kinetic isotope effect of 2.34 was seen for C–H insertion with gold catalysis.

**Scheme 24:**
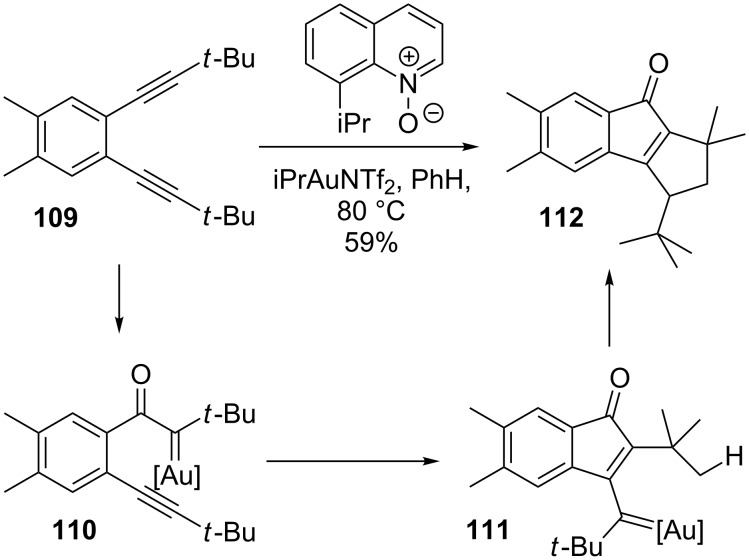
Gold carbene/alkyne cascade.

Hashmi demonstrated the viability of dual gold catalysis for carbene/alkyne cascades with diynes like **109**, which gave products from either a 1,2-methyl shift (not shown) or a C–H bond insertion to form enone **112** (Scheme 24) [97]. Though this report provided proof of principle, it focused on the generation of fused polycyclic products.

**Scheme 25:**
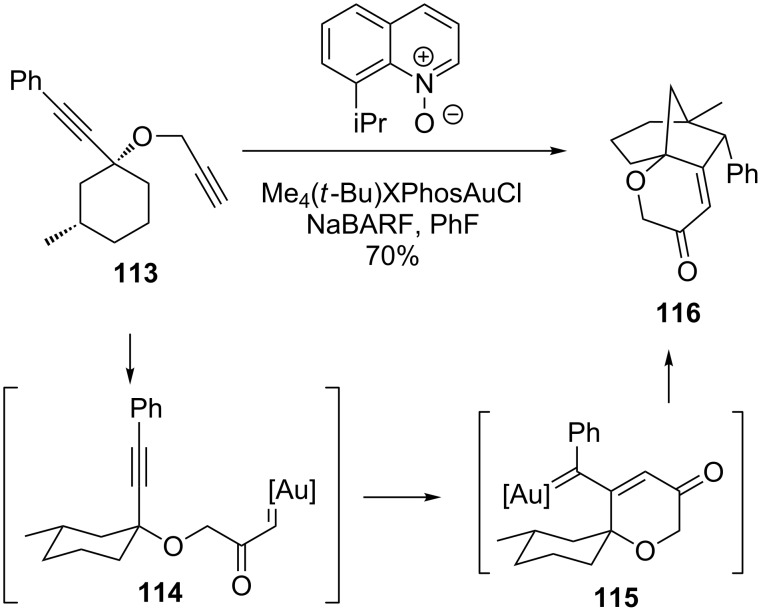
Gold carbene/alkyne cascade with C–H bond insertion.

Zheng demonstrated that an alkyne cascade could generate bridged polycyclic products like **116** from simple diyne precursors highly reminiscent of the May group’s substrates (Scheme 25) [98]. Here, a single gold species was used that contained a sterically bulky *t*-Bu-XPhos ligand. Many examples of the synthesis of ring-fused bridged bicycles were shown in a very nice demonstration of the reaction.

#### Platinum

**Scheme 26:**
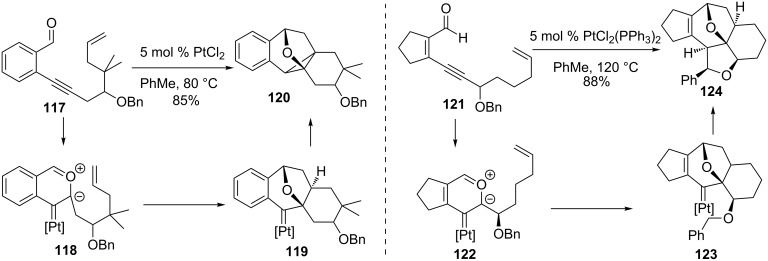
Platinum cascades.

Recent examples have used less common metals as catalysts for C–H bond insertion. Oh used platinum to generate intermediate 1,3-dipoles like **118** and **122**, which then undergo a cycloaddition to generate **119** or **123**, respectively (Scheme 26) [99–101]. Here, the reactivity of those carbenes diverges. Carbene **119**, which has a distal benzyl ether, undergoes a methine C–H bond insertion to form the caged cyclopropyl ring system in **120**. Alternatively, the proximity of the methylene of the benzyl ether in **123** allows for a benzylic C–H insertion to generate the bridged polycycle **124**.

#### Tungsten

**Scheme 27:**
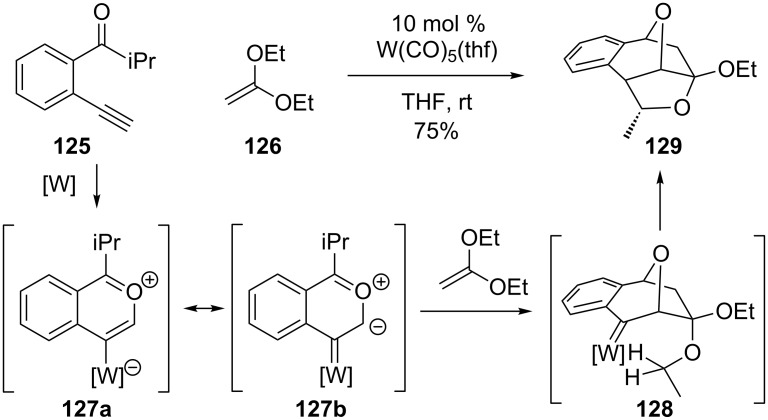
Tungsten cascade.

Iwasawa demonstrated a similar strategy for bridged-polycycle synthesis using a tungsten catalyst (Scheme 27) [102–103]. Again, the formation of a 1,3-dipole (see **127**) allows for a cycloaddition, though this example is intermolecular in nature. One of the ethereal ethyl groups in **128** is consequently poised for C–H bond insertion by the tungsten carbene to give **129** as a single diastereomer.

## Conclusion

Many of the early examples of bridged-polycycle synthesis via C–H bond insertion arose from strategies targeting the total synthesis of natural products. The results of these early efforts led to useful reaction conditions, a better understanding of stereoelectronic effects involved in the insertions, and inspiration for subsequent efforts of greater complexity. The transannular C–H bond insertion by an axially disposed carbene group that is needed for bridged-ring formation occurs readily, but pathways that lead to fused products having less ring strain or that lead to dimer formation must be excluded by catalyst control or reaction protocols (e.g., slow addition of substrate). As a result of the studies reported herein, many representative core systems for natural products have been synthesized, and our understanding of substrate control is becoming much better defined. In the future, greater catalyst control for these reactions should be pursued. Ideally, the catalyst would dictate the diastereoselectivity, the enantioselectivity, and the location of C–H bond insertion to provide any of the possible product isomers selectively on demand.
